# Open versus robotic-assisted repair of midline ventral hernias with defect width 2–8 cm – a randomized clinical trial (OVER)

**DOI:** 10.1007/s10029-026-03617-7

**Published:** 2026-04-02

**Authors:** Kristian Als Nielsen, Alexandros Valorenzos, Eirini Tsigka, Cathrine Häbel Frandsen, Per Helligsø, Sofie Ronja Petersen, Mark Bremholm Ellebaek, Michael Festersen Nielsen

**Affiliations:** 1 Department of General Surgery, University Hospital of Southern Denmark, Aabenraa, Denmark; 2 Department of Regional Health Research, University of Southern Denmark, Aabenraa, Denmark; 3 Department of Clinical Research, University Hospital of Southern Denmark, Aabenraa, Denmark; 4 Department of General and Plastic Surgery, Sygehus Lillebaelt, Vejle, Denmark; 5 Department of Plastic Surgery, Aarhus University Hospital, Aarhus, Denmark; 6 Department of Clinical Research, Faculty of Health Sciences, University of Southern Denmark, Odense, Denmark; 7 Research Unit of Surgery, Odense University Hospital, Odense, Denmark; 8 Department of Surgery, Horsens Regional Hospital, Horsens, Denmark

**Keywords:** Robotic-assisted surgery, Ventral hernia repair, Randomized controlled trial, Length of stay, Quality of life, Open repair

## Abstract

**Purpose:**

To compare short-term clinical outcomes, inflammatory response, and patient-reported quality of life after open versus robotic-assisted ventral hernia repair (oVHR vs. rVHR).

**Methods:**

This randomized controlled trial included adults with midline ventral hernias (2–8 cm). Patients were randomized to oVHR or rVHR. The primary endpoint was postoperative length of stay (LOS). Secondary outcomes included operative time, complications, quality of life, and inflammatory response measured by CRP. The primary outcome was analyzed using Poisson regression adjusted for defect size, repeated measures with linear mixed-effects models, and binary outcomes with logistic regression.

**Results:**

Fifty-six patients were analyzed (rVHR = 29, oVHR = 27). Median operative time was longer for rVHR (129 min) than oVHR (80 min, p < 0.001). Mean LOS was significantly shorter after rVHR (0.46 days, 95 % CI 0.21–0.72) than oVHR (1.96 days, 95 % CI 1.43–2.49, p < 0.001). The benefit increased with defect size, corresponding to a predicted one-day difference at 17 cm². Postoperative CRP levels were lower after rVHR on both day 1 (23 vs 46 mg/L, p < 0.001) and day 3 (33 vs 70 mg/L, p < 0.001). Complication rates were similar (21 % vs 26 %, p = 0.6). Quality-of-life improvements at 3 and 6 months were comparable between groups.

**Conclusion:**

Robotic-assisted ventral hernia repair was associated with shorter hospitalization particularly for larger defects. The surgical stress response was also significantly lower following rVHR, but with longer operative time and no differences in complications or patient-reported outcomes.

ClinicalTrials.gov NCT05906017, registered May 23 2023.

## Introduction

Open ventral hernia repair (oVHR) has been the standard approach for many years, offering a well-established method for addressing both simple and complex hernias [1, 2]. In recent years, the use of robotic-assisted ventral hernia repair (rVHR) has increased exponentially and emerged as a promising alternative, with the potential to improve clinical outcomes through enhanced precision and dexterity, reduced surgical trauma, and faster recovery [3, 4].

The laparoscopic approach to ventral hernia repair has been employed for decades, providing the typical benefits of minimally invasive surgery, including reduced surgical trauma, a lower risk of surgical site occurrences, and shorter hospital stay compared to the open approach [5, 6]. However, concerns have emerged regarding severe long-term complications due to the intraperitoneal mesh placement including chronic pain associated with mesh fixation [7–9]. Although transabdominal laparoscopic placement of mesh in the preperitoneal or retro-muscular space is technically achievable, the procedure requires advanced surgical skill and is time-consuming, especially when component separation is required [7, 8, 10, 11].

The introduction of robotic platforms in ventral hernia surgery has advanced the field by enabling minimally invasive techniques that combine the benefits of traditional open surgery with the advantages of laparoscopic methods. By enhancing instrument dexterity, these systems allow for intricate maneuvers and enable mesh placement in anatomical planes that are often inaccessible with conventional laparoscopy and would typically necessitate open surgery [12, 13].

As rVHR gains popularity, it is essential to rigorously evaluate its clinical outcomes compared to the alternatives to justify the substantial investment in a robotic platform [14]. While the initial investment and ongoing maintenance and instrument costs of the robotic systems are significant, it has been proposed that savings from potentially reduced hospital stays, lower complication rates, and faster patient recovery could offset these expenses [15].

A recent systematic review found that rVHR was associated with a shorter hospital stay (3.16 vs. 7.41 days), fewer complications and longer operative times compared with oVHR [16]. However, the evidence was highly heterogeneous, with marked variation in surgical techniques, definitions, study design, and follow-up, limiting the reliability and generalizability of the findings. The only randomized controlled trial to date comparing robotic and open retromuscular repair found no difference in overall complications, but a significantly shorter length of stay after rVHR (1 vs. 2 days) [17].

High-quality data on this subject remain limited. To address this gap, the present study provides a comprehensive evaluation of short-term outcomes.

### Objectives

The primary objective of this study is to determine whether rVHR for both primary and incisional midline, medium to large-sized ventral hernias is associated with a shorter postoperative LOS, compared to oVHR.

Our hypothesis is that the postoperative LOS for patients undergoing rVHR will be shorter than for those undergoing oVHR.

Additionally, the study aims to evaluate whether rVHR is linked to a reduced risk of postoperative complications, enhanced postoperative quality of life, and a diminished surgical stress response compared to oVHR. We furthermore want to identify the patient subgroup for whom robotic-assisted surgery provides the greatest clinical advantage in our setting.

### Methods

#### Study design

We performed a pragmatic, multicenter, non-blinded, parallel randomized controlled trial (RCT) comparing open versus robotic-assisted ventral hernia repair. The trial was approved by the regional ethics committee (approval number: S-20220106) and registered at ClinicalTrials.gov (registration number: NCT05906017) on May 23, 2023. The study was conducted in accordance with the principles of the Declaration of Helsinki and adheres to the CONSORT guidelines for reporting randomized trials.

A detailed trial protocol describing the study methodology has been published and is registered with the Open Science Framework (OSF) [18]. Participants were randomized to undergo either open or robotic-assisted ventral hernia repair. The study was conducted at the University Hospital of Southern Denmark, Aabenraa and Odense University Hospital, Svendborg. Surgical procedures were performed between May 2023 and April 2025, and the 6-month follow-up of the last patient was completed in October 2025.

### Participants

Patients eligible for this study were evaluated in the outpatient clinic, where a clinical assessment was performed. If a midline ventral hernia was evident upon physical examination, patients were informed about the study and provided with detailed informational materials. Written informed consent was obtained before inclusion in the trial. Participants retained the right to withdraw their consent at any time. Smokers were encouraged to cease smoking at least six weeks before surgery, although this was not mandatory. Likewise, overweight patients were advised to lose weight prior to surgery.

### Eligibility criteria

Patients were eligible for inclusion if they were 18 years of age or older, had an American Society of Anesthesiologists (ASA) physical status classification of I–III, and had a clear clinical or radiological diagnosis of a midline ventral hernia - either primary (umbilical or epigastric) or incisional. The hernia defect width had to be between 2 and 8 cm based on clinical evaluation, including preoperative physical examination and/or imaging. Patients also had to be deemed suitable for elective surgery following anesthesiologic assessment. Additional inclusion criteria were the ability to understand both written and spoken Danish and the provision of written informed consent.

Patients were excluded if they presented with an incarcerated ventral hernia requiring emergency surgery or if they were pregnant at the time of enrollment. Additional exclusion criteria were presence of recurrent midline hernia, chronic pain conditions requiring regular analgesic medication (e.g., paracetamol, NSAIDs, or opioids), active malignancy, or a history of psychiatric or substance use disorders likely to interfere with trial participation and follow-up compliance. Patients with co-existing inflammatory or immunological diseases requiring medical treatment were also excluded, as were those with a body mass index (BMI) exceeding 40 kg/m².

### Randomization and treatment allocation

To ensure balanced group allocation, block randomization with a block size of six was used, and patients were stratified according to estimated defect diameter (2–4 cm, 4–6 cm, and 6–8 cm) before assignment to either rVHR or oVHR. Randomization was performed using Research Electronic Data Capture (REDCap) with a computer-generated allocation sequence created and accessible only to an independent data manager from the Open Patient data Explorative Network (OPEN). Neither the project manager nor the personnel enrolling participants and assigning interventions had access to the randomization code.

### Important changes after trial commencement

Several changes were made to the trial after commencement due to recruitment challenges and in order to reflect real-world clinical practice more accurately:

### Change of primary outcome

The primary outcome was changed from the Abdominal Hernia Questionnaire (AHQ) to LOS. This decision was made after it became evident that the initially planned sample size (110 patients) could not be achieved within the available timeframe. A revised power calculation was performed using LOS as the new primary outcome, based on effect size estimates derived from a recent database study comparing robotic and open ventral hernia repair [19]. This allowed for a reduced sample size of 60 patients, making the trial feasible within the planned period. LOS was selected as the primary endpoint because it provides a somewhat objective and clinically meaningful measure of postoperative recovery, linked to patient well-being, complication burden, and hospital resource use [20–23].

### Expansion of inclusion criteria

Initially, only patients with a BMI ≤ 35 were eligible. This threshold was later increased to BMI ≤ 40 to improve recruitment and better reflect the real-world population undergoing ventral hernia repair.

Furthermore, the study initially included only patients with primary ventral hernias (PVH). During the recruitment phase, patients with incisional hernias (IH) were also included to increase generalizability and achieve adequate sample size.

### Intervention

In most cases, the techniques described below were employed, but depending on the size of the hernia as well as patient and surgeon preferences, alternative methods such as a pre-peritoneal mesh placement, “mini-stoppa” or onlay mesh placement were utilized.

Both interventions were performed by skilled surgeons proficient in both techniques under general anesthesia, with patients fasting for at least 6 h prior to the procedure. In most cases, antibiotic prophylaxis was administered. Pain management was standardized across both groups and involved a combination of systemic analgesics and local anesthetic administered at the incision sites (bupivacaine 0.5%). To avoid influencing the systemic inflammatory response, no corticosteroids or NSAIDs were administered peri-operatively. In selected large open hernia cases, an epidural catheter was placed based on anesthesiologic assessment.

Patients were admitted on the day of surgery and were discharged based on their recovery and clinical criteria. Follow-up visits were scheduled at 1 and 3 days postoperatively to conduct blood tests and physical examination.

### oVHR intervention description (Rives-Stoppa technique)

The patient was placed in a supine position on the operating table. A midline incision was made over the hernia site, extending as necessary to fully expose the hernia defect. The subcutaneous tissue was dissected to reveal the anterior rectus sheath, which was then incised on both sides of the midline, allowing access to the rectus abdominis muscles. These components were retracted laterally to expose the posterior rectus sheath. The posterior rectus sheath was then carefully dissected away from the rectus muscles, creating a retromuscular space that extends laterally as well as cranially and caudally beyond the hernia defect, providing ample area for mesh placement and overlap. This is allowing the rectus muscle to widen and further medialize the linea alba, offsetting the tension at the suture line during midline abdominal wall reconstruction.

The defect in the posterior rectus sheath was closed with an absorbable polydioxanone 2.0 suture, and a large prosthetic flat mesh (polypropylene) is placed into the retromuscular space. The mesh should be large enough to cover the hernia defect and extend well beyond it in all directions to ensure wide overlap with healthy tissue. The mesh was secured with sutures to the posterior rectus sheath.

The anterior rectus sheath was then sutured back together in the midline, restoring the integrity of the abdominal wall. Finally, the skin and subcutaneous tissues were closed in layers [24].

### rVHR intervention description

The robotic repair was performed using da Vinci Xi^®^ system (Intuitive Surgical Inc., Sunnyvale CA, USA). Patients were placed in a supine position under general anesthesia. Pneumoperitoneum was established with CO₂ insufflation to 12 mmHg, and an 8-mm camera port along with two 8-mm robotic working ports were inserted laterally to the edge of the rectus compartment on one side of the abdomen. The robot was then docked, with its arms aligned to the working ports.

Sitting at the console, the surgeon incised the peritoneum to enter the preperitoneal plane, followed by a longitudinal incision of the posterior rectus sheath to access and develop the retrorectus space bilaterally. After complete dissection from the one lateral edge of the rectus compartment to the other, the hernia sac was reduced and the anterior fascial defect closed with a barbed absorbable V-LOC™ (Medtronic, Inc., Minneapolis, MN, USA) suture. A ProGrip™ self-fixating mesh (Medtronic Inc., Minneapolis, MN, USA), tailored to fit the retrorectus space, was positioned in the appropriate plane, and the posterior rectus sheath was closed to restore the posterior layer.

The robot was then undocked, the ports removed, and the skin incisions closed with absorbable sutures.

### Outcomes

The primary outcome of this trial was LOS measured in hours and converted to days, starting from the admission on the day of surgery until discharge.

Several secondary outcomes were assessed to provide a comprehensive evaluation of perioperative and postoperative differences between the two surgical approaches.

The systemic inflammatory response was evaluated by measuring plasma C-reactive protein (CRP) levels (mg/L) preoperatively and on postoperative days 1 and 3.

Perioperative data included estimated intraoperative blood loss (mL) and hernia defect size, which was measured intraoperatively according to the European Hernia Society (EHS) classification and transformed into defect area in cm^2^ using the formula for the area of an ellipse [25]. Surgical time was recorded to assess procedural efficiency.

Intraoperative and postoperative complications were systematically recorded, with postoperative complications classified according to the Clavien–Dindo (CD) grading system [26, 27].

Patient-reported outcomes were assessed using a modified version of the AHQ, administered preoperatively and at 3 and 6 months postoperatively to evaluate health-related quality of life and symptom burden [28, 29].

AHQ scores were calculated as the mean of the responses to each question (range 1–4), with higher scores indicating better hernia-related quality of life. A total score was made preoperatively and at 3 and 6 months postoperatively. In addition, scores were analyzed across two predefined domains - Physical and Appearance - based on the factor structure established in the original validation study [28, 30]. Pain was both analyzed as a part of the physical domain and separately as an individual domain.

### Sample size and statistical analysis

Statistical analyses were conducted using R (version 4.3.3) and stata BE (version 19).

A priori power calculations were conducted using Monte Carlo simulations based on a Poisson regression model for the primary outcome (LOS), assuming complete follow-up. It was expected that patients undergoing robotic-assisted ventral hernia repair (rVHR) would have a mean LOS of 0.5 days, compared to 2.1 days for those undergoing open ventral hernia repair (oVHR), based on national registry data by Henriksen et al. [19]. With an alpha level of 0.05, a sample size of 20 patients per group was estimated to achieve a power greater than 90%. To account for potential dropout and missing data, we aimed to enroll 60 participants.

Descriptive statistics were used to summarize baseline characteristics, intraoperative findings, and secondary outcomes. Continuous variables were reported as mean with standard deviation or median with interquartile range, depending on distribution. Categorical variables were reported as counts and percentages.

The primary outcome, length of hospital stay, was analyzed using a Poisson regression model. Hernia defect size was included as a covariate, and subgroup analyses were performed to explore potential effect modification by defect size using interaction terms and likelihood ratio tests. We validated the fit of the model by assessing residual plots and the over-dispersion parameter.

Repeated outcome measures, including health-related quality of life (AHQ) and CRP, were analyzed using linear mixed-effects models with random intercepts to account for intra-individual correlation over time. Bootstrapped confidence intervals and p-values were derived to overcome the missed assumption on normality. Treatment effects over time were assessed using likelihood ratio tests to control for inflated type I error.

Logistic regression was used to estimate odds ratios (ORs) with 95% confidence intervals for binary outcomes such as the occurrence of any postoperative complication within 6 months postoperatively. Due to the low number of events, no formal statistical comparisons were performed for rare outcomes including readmission, reoperation, hernia recurrence, hematoma, seroma, urinary retention, wound infection, and chronic pain at 3 months. The limited event rates also precluded statistical comparison of Clavien–Dindo complication grades. All regression models were validated graphically by residual plots in terms of their assumptions.

Further details on the statistical analysis plan and power calculations are available in the published study protocol [18].

### Subgroup analysis

To investigate whether the effect of surgical approach (rVHR vs. oVHR) on LOS differed according to hernia type – IH versus PVH – a two-way interaction term (group × hernia type) was included in the linear mixed-effects model. Marginal means and the *p*-value for the interaction term were examined to explore the nature of the interaction. The same analytical approach was applied to assess potential effect modification by mesh placement both on LOS (group×mesh placement) and CRP (time×group×mesh placement).

## Results

### Baseline and intraoperative findings

**Fig. 1:**
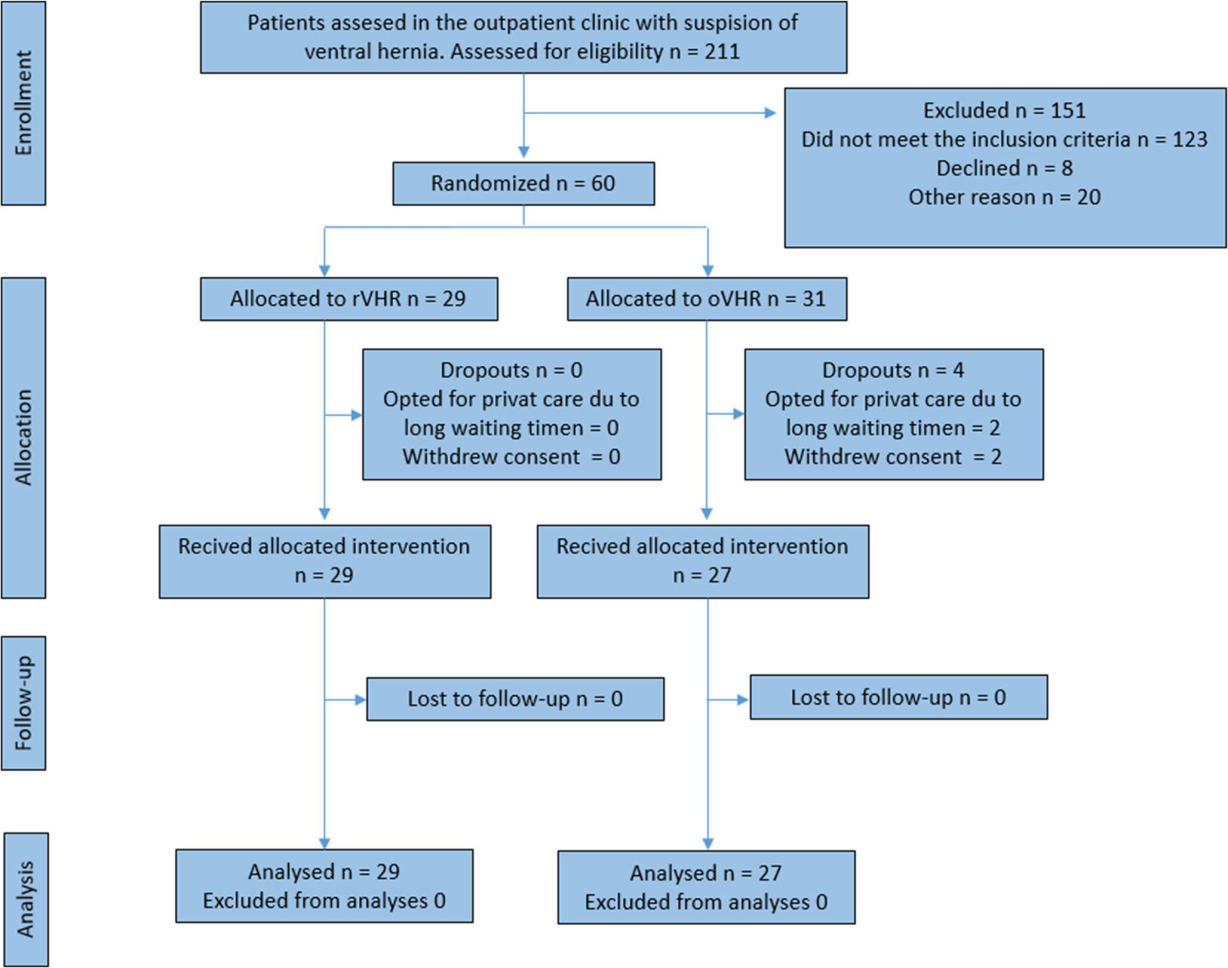
CONSORT diagram of patient allocation and follow-up for primary outcome

A total of 60 patients were randomized for the trial. Of these, 2 patients opted for private treatment and 2 withdrew consent before receiving their allocated treatment, leaving 29 patients in the rVHR group and 27 patients in the oVHR group for intention to treat analysis of the primary outcome (Fig. 1).

As shown in Table 1, the median age was 62 years in the rVHR group and 63 years in the oVHR group. Mean BMI was similar across groups (31.8 vs. 31.5), as were ASA classifications and comorbidity burden measured using the Charlson Comorbidity Index. Distribution of sex, hernia location, as well as alcohol and smoking habits were also comparable between groups.

**Table 1:** Preoperative patient and hernia characteristics

Variables	rVHR (*n* = 29)	oVHR (*n* = 27)
Patient demographics:		
Age (years), median (IQR)	62 (46,71)	63 (55,69)
Male sex	19 (66%)	17 (63%)
BMI (kg/m^2^), mean (SD)	31.8 (4.5)	31.5 (4.7)
*Smoking status*		
Never	13 (45%)	9 (33%)
Former	6 (21%)	7 (26%)
Active	10 (34%)	11 (41%)
*Alcohol consumption*		
Never	2 (6.9%)	0 (0%)
Rarely	6 (21%)	9 (33%)
1–10 units per week	19 (66%)	17 (63%)
> 10 units per week	2 (6.9%)	1 (3.7%)
Comorbidity and risk scores		
*ASA grade*		
I	3 (10%)	1 (3.7%)
II	17 (59%)	16 (59%)
III	9 (31%)	10 (37%)
Charlsons co-morbidity index, median (IQR)	2.0 (0.5, 3.5)	2.0 (1.0, 3.0)
*Comorbidities*		
Hypertension	13 (45%)	12 (44%)
Heart disease	6 (21%)	3 (11%)
Lung disease	8 (28%)	3 (11%)
Diabetes mellitus	4 (14%)	4 (15%)
Dyslipidemia	2 (6.9%)	5 (19%)
Previous abdominal surgery	13 (45%)	11 (41%)
Hernia characteristics		
Epigastric	4 (14%)	4 (15%)
Incisional	5 (17%)	6 (22%)
Umbilical	20 (69%)	17 (63%)

Data are reported as median (IQR), mean (SD), or number (%), as appropriate

Intraoperatively measured defect size did not differ significantly between groups (Table 2). A significant difference was observed in mesh positioning: 93% of patients in the rVHR group received retromuscular mesh placement compared to 59% in the oVHR group (*p* = 0.002), while onlay mesh (30%) and non-mesh repair (7%) was used exclusively in the oVHR group. Median mesh size was significantly larger in the rVHR group (225 cm² vs. 150 cm²; *p* = 0.006). Operative time was also significantly longer in the rVHR group (median 129 vs. 80 min; *p* < 0.001).

**Table 2:** Intraoperative outcome

Variables	rVHR	oVHR	*p*-value
Estimated blood loss (mL), median (IQR)	15.0 (10.0,22.5)	17.5 (2.25,61.25)	0.6 ^β^
Mesh size (cm^2^), median (IQR)	225 (180, 240)	150 (36, 225)	0.006 ^β^
Intraoperative hernia defect size (cm^2^) median (IQR)	9 (6, 14)	7 (3, 28)	0.8 ^β^
Procedure time (min), median (IQR)	129 (115,151)	80 (44,101)	<0.001 ^β^
Antibiotic prophylaxis, n (%)	18 (62%)	19 (70%)	0.58 ^β^
Mesh Placement			
No mesh used	0 (0%)	2 (7.4%)	0.004 ^φ^
Onlay	0 (0%)	8 (30%)	
Pre peritoneal	2 (6.9%)	1 (3.7%)	
Retro rectus	27 (93%)	16 (59%)	
Intraoperative complications			
Bowel injury	0 (0%)	2 (7.4%)	-
Drain placement	0 (0%)	11 (41%)	-
Epidural catheter placement	0 (0%)	9 (33%)	-

Data are reported as median (IQR) or number (%), as appropriate. Between-group comparisons were performed using the Wilcoxon rank sum test β for non-normally distributed continuous variables and the Fisher’s exact test φ for categorical variables.

Median estimated blood loss was comparable (15 ml in rVHR vs. 17.5 ml in oVHR; *p* = 0.6). Two minor bowel lesions occurred in the oVHR group but did not require further intervention other than oversewing. Drains were placed in 41% of patients in the oVHR group and in none of the rVHR patients. Similarly, epidural catheters were placed in 33% of oVHR patients and in none of the rVHR cases. Antibiotic prophylaxis was administered to 70% of patients in the oVHR group and 62% in the rVHR group (*p* = 0.58).

### Length of stay

**Fig. 2:**
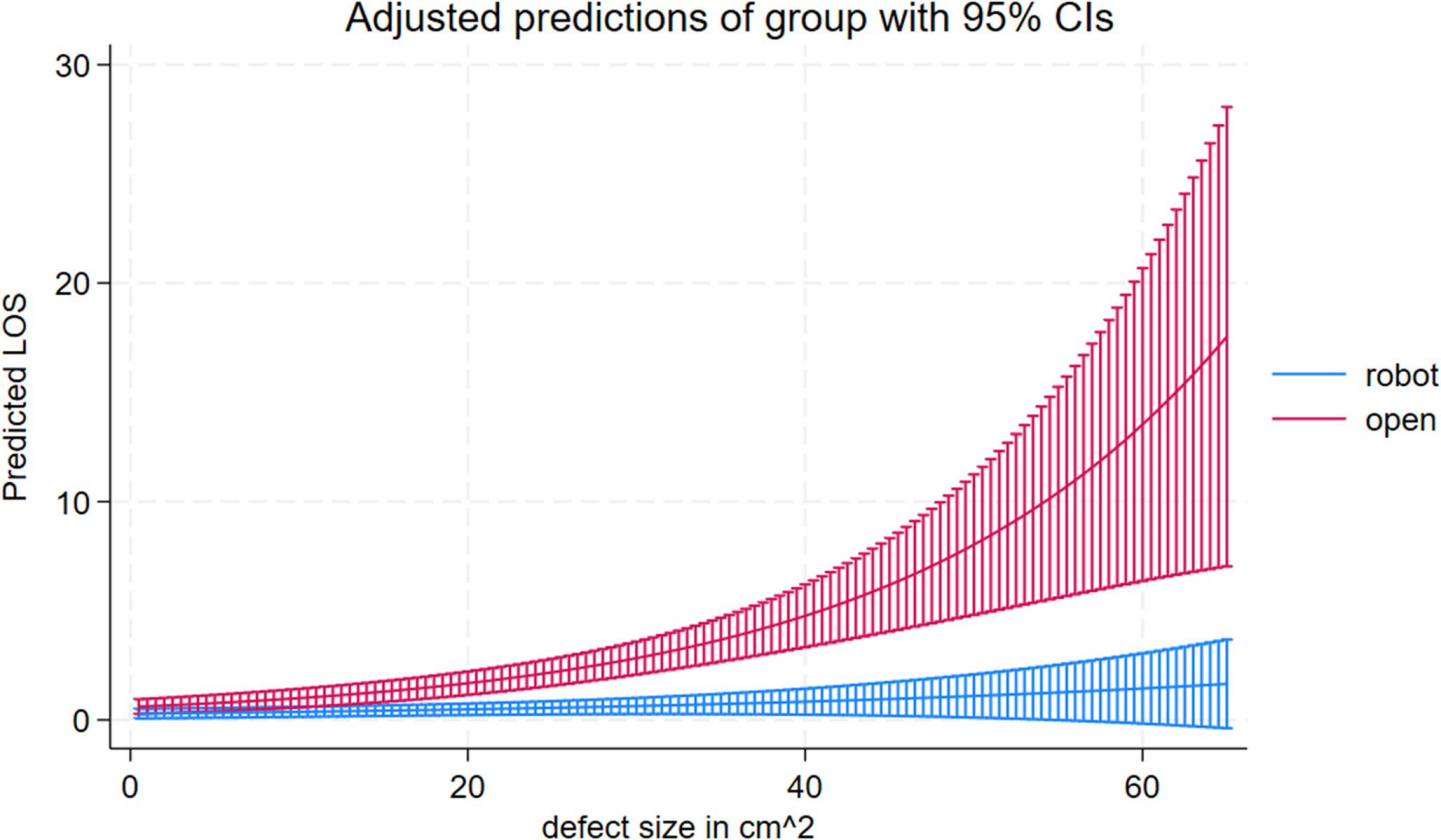
Predicted length of stay by defect size and surgical approach

The mean LOS was significantly shorter in the rVHR group compared to the oVHR group: 0.46 days (95% CI: 0.21–0.72) vs. 1.96 days (95% CI: 1.43–2.49), *p* < 0.001. Figure 2 illustrates the modeled interaction between hernia defect size and LOS. In the rVHR group, LOS increased by 1.59% per cm² increase in defect size, while in the oVHR group, the corresponding increase was 6.12%, *p* < 0.001. The predicted LOS in the rVHR group followed a nearly linear trajectory, whereas the oVHR group shows an exponential increase in LOS with larger defect sizes. According to the model, a difference of one full day in predicted LOS between the two surgical approaches was observed at a defect size of approximately 17.1 cm².

The subgroup analysis showed that the effect of surgical approach on LOS did not differ significantly between patients with incisional and primary ventral hernias (*p* = 0.46). In contrast, mesh placement significantly modified the difference in LOS between groups. After controlling for defect size, the mean LOS was 0.50 days (95% CI, 0.23 to 0.78) for rVHR, 0.21 days (95% CI, 0 to 0.62) for oVHR with onlay placement, and 2.60 days (95% CI, 1.86 to 3.35) for oVHR with retromuscular placement.

### Quality of Life – Abdominal hernia questionnaire

There were no statistically significant differences between the rVHR and oVHR groups in patient-reported quality of life across any of the assessed domains, including physical symptoms, appearance, and pain (Table 3). A total of 54 of 56 patients (96.4%) completed the questionnaire; one patient died before complete follow-up and one did not respond.

**Table 3:** Mean Abdominal Hernia-Q (AHQ) scores by domain with 95% confidence intervals at baseline, 3 months, and 6 months postoperatively for rVHR and oVHR

AHQ-domain	Group	Baseline score (95% CI)*	3 month score (95% CI)	6 month score (95%CI)	*p*-value
Total	rVHR	23.66 (22.64;24.68)	40.35(38.90;41.80)	40.39(38.78;41.99)	0.34
	oVHR	23.66(22.64;24.68)	39.85(37.59;42.12)	41.85(40.26;43.43)	
Physical	rVHR	16.86(16.26;17.45)	22.33(21.47;23.19)	22.31(21.48;23.15)	0.27
	oVHR	16.86(16.26;17.45)	22.11(21.06;23.15)	23.21(22.31;24.12)	
Appearance	rVHR	6.80(6.12;7.48)	18.12(17.21;19.03)	18.13(17.22;19.04)	0.71
	oVHR	6.80(6.12;7.48)	17.64(16.11;19.17)	18.56(17.50;19.62)	
Pain	rVHR	3.27(3.10;3.43)	3.79(3.57;4.00)	3.73(3.53;3.93)	0.69
	oVHR	3.27(3.10;3.43)	3.77(3.51;4.03)	3.87(3.66;4.07)	

* To account for baseline differences and improve comparability between groups, baseline values were constrained to be equal eliminating systematic group differences between the groups

### CRP

Postoperative levels of CRP were significantly higher in the oVHR group compared to the rVHR group at both postoperative day 1 and day 3. On day 1, the mean CRP was 22.92 mg/L in the rVHR group versus 46.41 mg/L in the oVHR group. On day 3, CRP levels increased to 32.57 mg/L in the rVHR group and 69.70 mg/L in the oVHR group. These differences were statistically significant (*p* < 0.001), indicating a more pronounced systemic inflammatory response following open repair. Baseline values were constrained to be equal eliminating systematic group differences between the groups.

Effect-modification analysis by mesh placement showed a marked difference in the postoperative inflammatory response. After stratifying the open repairs into onlay and retromuscular techniques and adjusting for defect size, open onlay repair demonstrated lower CRP levels than both robotic retromuscular repair and open retromuscular repair. On postoperative day 1, mean CRP was 17.9 mg/L (95% CI, 10.7 to 25) after open onlay repair compared with 24.5 mg/L (95% CI, 18.3 to 30.7) after robotic retromuscular repair and 62 mg/L (95% CI, 46.1 to 77.9) after open retromuscular repair (*p* < 0.001). A similar pattern persisted on day 3 (21.5 mg/L vs. 34.7 mg/L vs. 93.8 mg/L) (95% CI 13.1 to 29.9 vs. 22.6 to 46.7 vs. 67.8 to 119.8).

### Postoperative findings

The overall complication rate did not differ significantly between groups, with 6 patients (21%) in the rVHR group and 7 patients (26%) in the oVHR group experiencing at least one complication (OR: 0.75, *p* = 0.60) (Table 4).

**Table 4:** Secondary postoperative outcome

Outcome	rVHR (*n* = 29)	oVHR (*n* = 27)	OR (95% CI)	*p*-value
Any complication	6 (21%)	7 (26%)	0.75 (0.22–2.59)	0.6
Readmission	1 (3%)	1 (4%)	-	-
Reoperation	1 (3%)	0 (0%)	-	-
Recurrence	1 (3%)	0 (0%)	-	-
Hematoma	1 (3%)	1 (4%)	-	-
Seroma	2 (7%)	1 (4%)	-	-
Urinary retention	1 (3%)	0 (0%)	-	-
Wound infection	1 (3%)	1 (4%)	-	-
Pain after 3 months*	1 (3%)	6 (22%)	-	-
Clavien-Dindo grade (*n* = 6 for rVHR and *n* = 2 for oVHR)				
I	4 (67%)	1 (50%)	-	-
II	0 (0%)	0 (0%)	-	-
IIIa	2 (33%)	1 (50%)	-	-

The proportion of patients experiencing at least one complication was compared between groups using logistic regression. Due to the low number of events, no formal statistical comparisons were performed for the remaining secondary postoperative outcomes or Clavien-Dino classification.* Pain reported after 3 months was considered a late outcome and not classified according to the Clavien–Dindo system. No Clavien–Dindo complications above grade IIIa were observed

A total of 7 surgical site occurrences were documented: one hematoma in each group, two seromas in the rVHR group and one in the oVHR group, as well as one wound infection in each group.

Only 8 of the postoperative complications could be classified using the Clavien-Dindo system, as chronic pain was not included in this classification. In the rVHR group, there were 4 grade I complications and 2 grade IIIa complications. In the oVHR group, there was one grade I and one grade IIIa complication. No severe complications (CD grade ≥IIIb) were observed in either group.

Pain at 3 months was the most commonly reported complaint, occurring in 6 patients (22%) in the oVHR group and 1 patient (3%) in the rVHR group. At 6-month follow-up, pain had resolved in 2 of the 6 oVHR patients who reported pain at 3 months but persisted in the remaining cases. Only one recurrence was observed, occurring in the rVHR group; no recurrences were reported in the oVHR group.

## Discussion

Our primary finding was a significantly shorter LOS following rVHR compared with oVHR, with a mean LOS of 0.46 days versus 1.96 days (*p* < 0.001). This difference was primarily driven by patients with larger hernia defects in the oVHR group: LOS increased by 1.6% per cm² in the rVHR group compared with 6.1% in the oVHR group (Fig. 2). These findings are consistent with previous studies, which generally favour rVHR, although the reported LOS ranges vary substantially across studies due to differences in study design, type of repair, and hospital discharge policies, making direct comparisons challenging [16, 17, 19, 31, 32].

In our population, robotic repair was not associated with any measurable benefits for small hernias across any outcome, including LOS (Fig. 2). However, for larger hernias, a clinically meaningful reduction in LOS was observed. When the defect size exceeded 17.1 cm², the predicted LOS difference reached one full day. A shorter length of stay not only reduces direct hospital costs but also improves patient flow and bed availability, thereby enhancing overall system efficiency. From a patient perspective, earlier discharge facilitates faster return to normal activities and work, reduces exposure to hospital-acquired complications, and is associated with higher patient satisfaction [20–23].

In line with clinical practice, smaller hernias in the oVHR group were often managed using onlay repair - a technique that involves a less extensive dissection than Rives-Stoppa, and that is generally associated with faster recovery and shorter LOS. The inclusion of such procedures may have reduced the overall mean LOS in the oVHR group and thus attenuated the observed differences between surgical approaches. While this introduces potential bias, it reflects real-world surgical decision-making and the flexibility allowed by current clinical guidelines [1, 2, 33]. Subgroup analyses stratified by mesh placement confirmed this pattern, showing that, after controlling for defect size, mean LOS was 0.21 days for open onlay repair, 2.60 days for open retromuscular repair, and 0.50 days for rVHR, underscoring that the benefit of rVHR is most pronounced in larger hernias where retromuscular mesh placement is required.

Operative time was substantially longer in the rVHR group, with skin-to-skin time averaging 49 min more than oVHR (*p* < 0.001). This difference likely reflects the more time-consuming dissection required for robotic retrorectus mesh placement and has also been reported in previous studies comparing robotic and open ventral hernia repair [17, 34, 35]. It may impact surgical cost efficiency and OR utilization, particularly in high-volume centers.

**Fig. 3:**
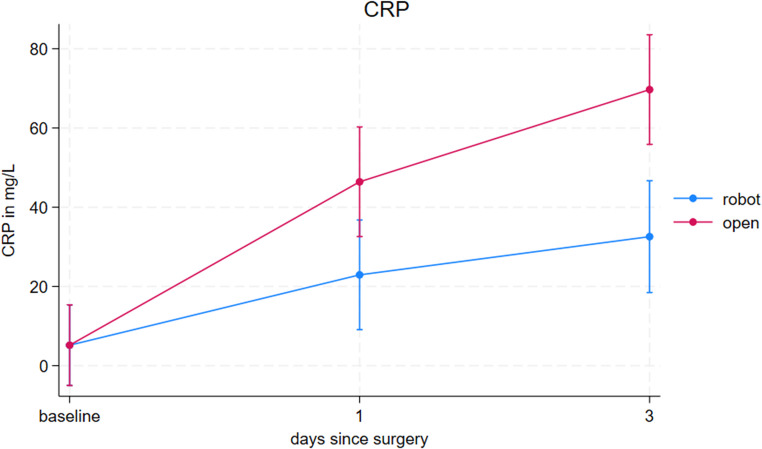
Postoperative C-reactive protein (CRP) levels following robotic-assisted (rVHR) and open ventral hernia repair (oVHR), measured preoperatively (baseline) and on postoperative days (POD) 1 and 3

Postoperative CRP levels were significantly lower in the rVHR group compared with the oVHR group, indicating a reduced systemic inflammatory response (Fig. 3). The magnitude of CRP release generally reflects the extent of tissue trauma, with less invasive procedures eliciting lower cytokine levels [36–38]. This pattern supports the notion that robotic-assisted repair represents a less invasive procedure. These findings align with previous randomized trials comparing robotic and conventional approaches across different procedures, all of which have demonstrated lower postoperative inflammatory markers following robotic surgery [37, 39, 40]. The subgroup analysis provided additional nuance. When the open procedures were stratified into onlay and retromuscular mesh placement, a markedly higher inflammatory response was observed after open retromuscular repair, whereas open onlay repair elicited an even lower CRP response than rVHR, mirroring the pattern observed in the LOS subgroup analysis. However, it remains uncertain whether these biochemical differences translate into meaningful clinical benefits beyond LOS.

Despite indications of reduced surgical invasiveness with rVHR, postoperative complication rates did not differ significantly between groups, with an overall incidence of 21% in the rVHR group and 26% in the oVHR group (OR: 0.75, *p* = 0.60). Postoperative complications were generally minor. Chronic pain at 3 months was the most frequently reported issue, occurring in 6 (22%) of patients in the oVHR group compared with only 1 (3%) in the rVHR group. At the 6-month follow-up, pain had resolved in two of the open cases but persisted in the remaining patients. Although the study was not powered to assess this outcome formally, the magnitude of the difference is clinically relevant and warrants further investigation. Only a small number of complications met the Clavien-Dindo criteria, and no severe complications (grade ≥ IIIb) were observed in either group. These findings are consistent with the only other randomized controlled trial comparing rVHR and oVHR by Warren et al. [17], who likewise reported no significant difference in overall or wound-related complications between robotic and open retromuscular repair (20% vs. 21%), and no differences in pain. However it contrasts with several previous observational studies reporting lower complication rates following robotic repair [19, 41]. The discrepancy may reflect differences in methodology and cohort characteristics. Notably, our study population consisted mainly of patients with medium sized hernias and relatively low surgical risk, which likely contributed to the overall low event rate of severe complications.

Both groups demonstrated a significant improvement in quality of life at 3 and 6 months postoperatively. Interestingly, no statistically significant differences were observed between groups in any domain of the AHQ, including total score, physical symptoms, cosmetic concerns, or pain, at either time point. These findings indicate that although rVHR was associated with reduced inflammatory response and shorter hospital stay, such objective benefits did not translate into measurable improvements in patient-reported quality of life within the 6-month follow-up period. Although the AHQ is a validated, hernia-specific instrument, it may lack the sensitivity to detect subtle differences between surgical approaches - particularly in a population with predominantly moderate sized hernia defects and low symptom burden at baseline. Both groups reported relatively high preoperative AHQ scores, and most patients provided near-perfect scores postoperatively across all domains. This ceiling effect likely limited the instrument’s ability to capture nuanced differences in patient experience and may have masked group-level differences in perceived outcomes. Other studies have similarly failed to demonstrate differences in long term patient reported quality of life between robotic and open hernia repair, although findings vary depending on follow-up duration, PROM instrument used, and population characteristics [17, 42, 43].

### Strengths and limitations

This study has several notable strengths, including its randomized controlled design, which ensured comparable baseline characteristics between groups and strengthens the causal interpretation of the observed differences. Moreover, the close postoperative follow-up with an almost complete response rate of PROMs further enhances data reliability. Nonetheless, certain limitations should be acknowledged. The inclusion of different surgical techniques within both treatment arms and the pooling of PVH and IH reflect real-world clinical practice and enhance external validity but may also introduce heterogeneity that reduces internal validity.

Although randomization balanced the distribution of PVH and IH between groups, pooling these entities could still have blurred the overall interpretation of outcomes, as they represent distinct pathophysiological conditions that may differ in patient comorbidity profiles, defect size, and postoperative recovery trajectories. As highlighted by Stabilini et al., such pooling has been critically questioned, since merging these populations may mask clinically relevant differences and reduce granularity in the assessment of treatment effects [44].

Nevertheless, this approach reflects routine clinical decision-making at our center, where the choice of repair is primarily determined by defect size rather than etiology, in accordance with Danish national guidelines [33]. Onlay and suture-only repairs remain accepted options for smaller defects in selected patients. This is particularly relevant for the population included in the present trial, comprising hernias with defect widths between 2 and 8 cm - a range that encompasses several clinically acceptable repair techniques. While these factors may introduce some heterogeneity and limit the ability to make direct comparisons between specific hernia types within the groups, they also strengthen the generalizability of the study by representing the real-world spectrum of surgical options applied to this patient group. Furthermore, our subgroup analysis showed that hernia type did not act as an effect modifier for LOS, meaning that the observed difference in LOS between rVHR and oVHR was consistent across both primary and incisional hernias. However, this finding should be interpreted with caution due to limited power, and a Type II error cannot be excluded.

The absence of blinding for patients and outcome assessors represents a methodological limitation that may introduce performance bias. However, all participating surgeons routinely performed both procedures and did not perceive either approach as experimental, making systematic behavioral differences unlikely. Likewise, patient discharge was based on standard clinical criteria, minimizing the risk of bias in LOS. Postoperative quality-of-life and pain outcomes were self-reported, and as the patients were not blinded, perception bias cannot be completely excluded.

Although the study was originally designed as a multicenter trial, only two patients were enrolled from a secondary site. As a result, the study functioned effectively as a single-center trial, which may limit its generalizability.

Finally, the sample size was insufficient to provide adequate statistical power for several of our secondary postoperative outcomes, including individual complications. As such, these results should be interpreted with caution, and further studies with larger cohorts are needed to draw definitive conclusions.

### Future directions

Future research comparing rVHR and oVHR should focus on larger and more complex hernias, where the clinical and economic advantages of the robotic approach, are likely to be most pronounced. Multicenter randomized trials with sufficient sample size to detect differences in secondary outcomes, distinguish between hernia types and surgical techniques, and incorporate blinded outcome assessment are warranted to validate these findings. Adequate follow-up duration will also be essential to assess long-term outcomes and hernia recurrence.

Importantly, future research should also aim to define a clinically and economically relevant threshold - such as a hernia defect size - above which robotic repair provides a net benefit. This may inform updated national recommendations on the selection of robotic versus open repair and could support more personalized surgical decision-making and support health economic models [45].

## Conclusion

Robotic-assisted ventral hernia repair was associated with a shorter length of stay, particularly in patients with larger hernia defects exceeding 17.1 cm². The surgical stress response, as measured by CRP, was significantly lower following rVHR. However, complication rates and patient-reported outcomes did not differ between groups, and operative time was significantly longer in the rVHR group.

## Data Availability

The datasets and statistical code are not publicly available due to ethical and legal restrictions under Danish data protection regulations. Requests may be considered by the corresponding author on a case-by-case basis and in accordance with applicable data sharing policies.
